# Supplementary material for: A versatile automated pipeline for quantifying virus infectivity by label-free light microscopy and artificial intelligence
Source: Nat Commun. 2024 Jun 15;15:5112. doi: 10.1038/s41467-024-49444-1 (PMC11180103; doi:10.1038/s41467-024-49444-1)
Supplement: Supplementary file 1 — Supplementary Information [file 41467_2024_49444_MOESM1_ESM.pdf]

Supplementary Tables

Supplementary table 1: Parameters of machine learning models used in grid search

Machine learning model	parameter	Value range
Gaussian Naïve Bayes (GNB)	var_smoothing	$\mathrm{numpy.logspace}(0, -9, \mathrm{num} = 100)$, equivalent to $\left\{10^{\frac{-9i}{99}} \middle| i = 0, 1, 2, \dots, 99\right\}$
Random Forrest Classifier (RF)		
Logistic Regression (LR)	solver	[“lbfgs”, “saga”]
	C	[1, 10]
Decision Tree Classifier (DT)	criterion	[“gini”, “entropy”]
	splitter	[“best”, “random”]
	max_depth	[5, 15, None]
Support Vector Machine (SVM)	kernel	[“linear”, “rbf”, “poly”]
	C	[1, 10]
k nearest neighbor (*k*-NN)	n_neighbors	[3, 5, 10]

Supplementary table 2: Performance metrics of machine learning models.

Accuracy indicates the fraction of correctly classified images, given by $\mathrm{Accuracy} = \frac{\mathrm{TP+TN}}{\mathrm{TP+TN+FP+FN}}$. The F1 score, also known as Sørensen–Dice coefficient, is the harmonic mean of precision and recall, given by $F1 = \frac{2 \cdot \mathrm{precision} \cdot \mathrm{recall}}{\mathrm{precision+recall}}$, where $\mathrm{precision} = \frac{\mathrm{TP}}{\mathrm{TP+FP}}$ and $\mathrm{recall} = \frac{\mathrm{TP}}{\mathrm{TP+FN}}$. The Matthews correlation coefficient (MCC) reflects the correlation between observed and predicted classes and is given by $\mathrm{MCC} = \frac{\mathrm{TP \cdot TN - FP \cdot FN}}{\sqrt{\mathrm{(TP+FP)(TP+FN)(TN+FP)(TN+FN)}}}$. The area under the receiver operator characteristic (ROC) curve (AUROC) is the integral of the function. TP = true positive, TN = true negative, FP = false positive, FN = false negative. Data indicate means ± standard deviation, n = 3

metric	SVM	*k*-NN	DT	GNB	LR	RF	DVICE
Accuracy	0.4246 ± 0.0044	0.6301 ± 0.0033	0.7473 ± 0.0143	0.7788 ± 0.0008	0.8848 ± 0.0018	0.8803 ± 0.0003	0.9912 ± 0.0012
F1 score	0.4279 ± 0.0073	0.5383 ± 0.0048	0.7386 ± 0.0126	0.7662 ± 0.0008	0.8837 ± 0.0016	0.8736 ± 0.0002	0.9912 ± 0.0012
AUROC	0.4353 ± 0.0056	0.5844 ± 0.0042	0.7879 ± 0.0232	0.8489 ± 0.0016	0.9404 ± 0.0027	0.9429 ± 0.0011	0.9914 ± 0.0012
MCC	-0.1190 ± 0.0077	0.1286 ± 0.0133	0.4522 ± 0.0307	0.5259 ± 0.0022	0.7550 ± 0.0039	0.7455 ± 0.0008	0.9825 ± 0.0024

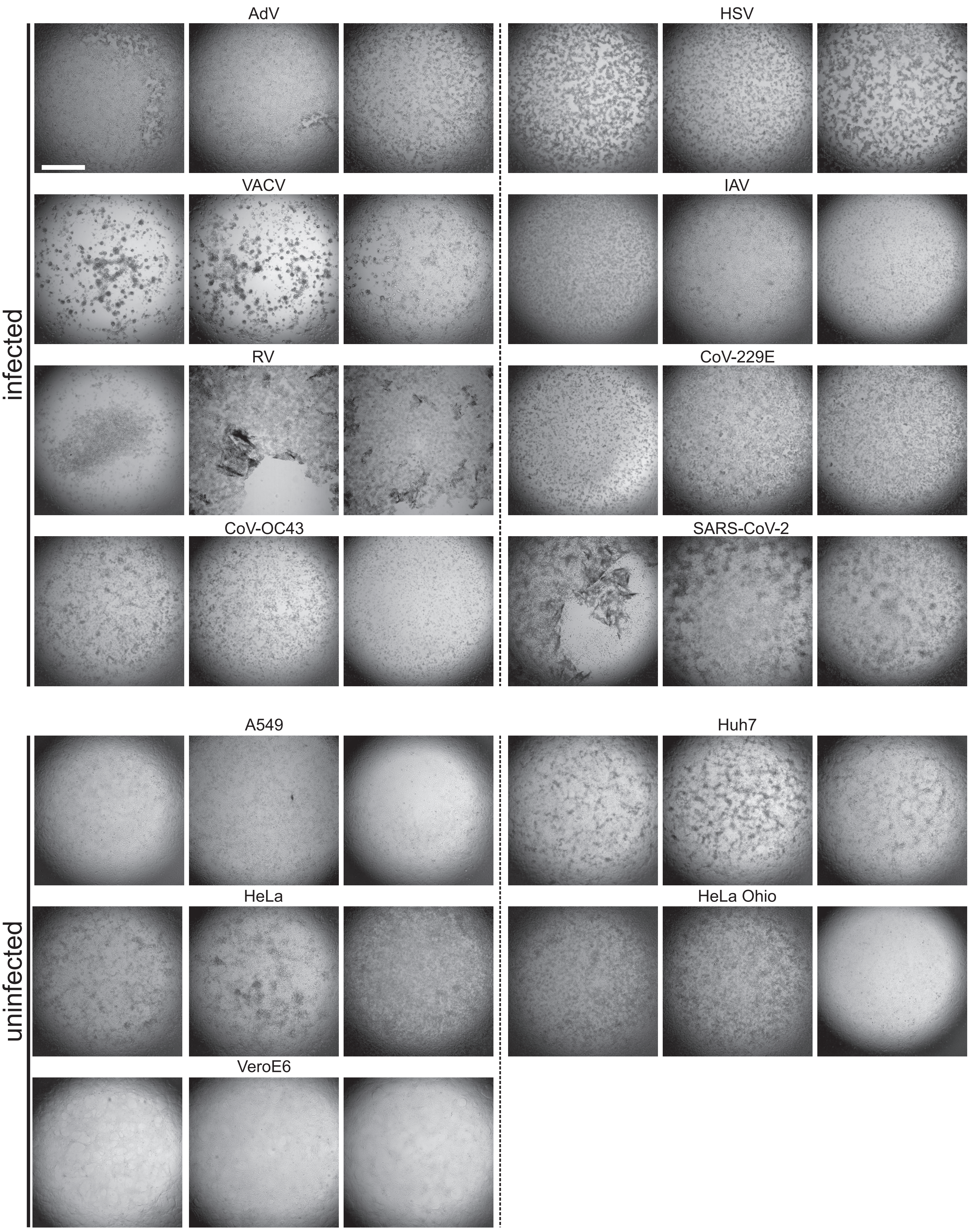

Figure S1: Examples of image dataset

Example images of infected and uninfected samples from the dataset. Scale bar = 1 mm.

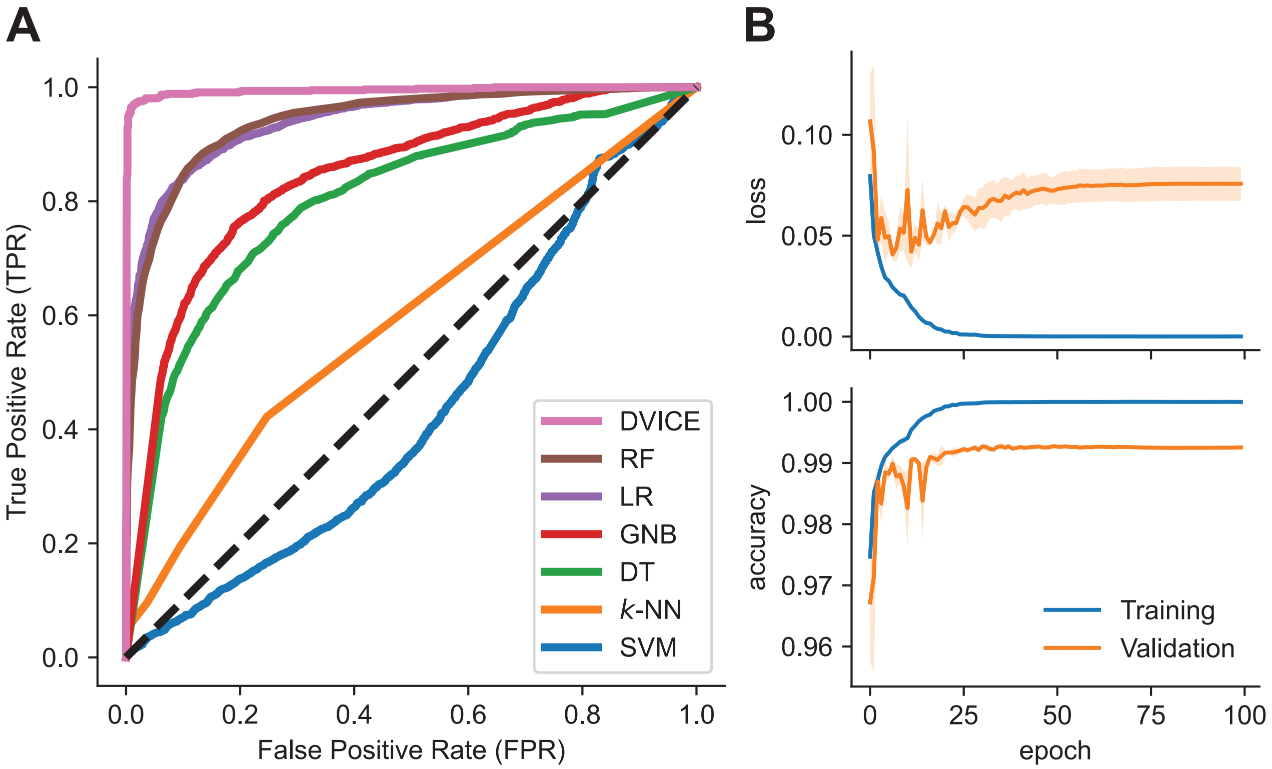

Figure S2: Performance characteristics of ML models and training behavior of DVICE

(A) Receiver operating characteristic (ROC) curves of ML models from Figure 2A. SVM = Support Vector Machine, LR = Logistic Regression classifier, GNB = Gaussian Naïve Bayes, RF = Random Forest classifier, DT = decision tree classifier, *k*-NN = *k*-nearest neighbors. The dotted line indicates a random classifier. **(B)** Graphs show binary cross entropy loss and accuracy of DVICE model on training and validation dataset. Shaded regions indicate standard deviation, n = 3. Source data are provided as a Source Data file.

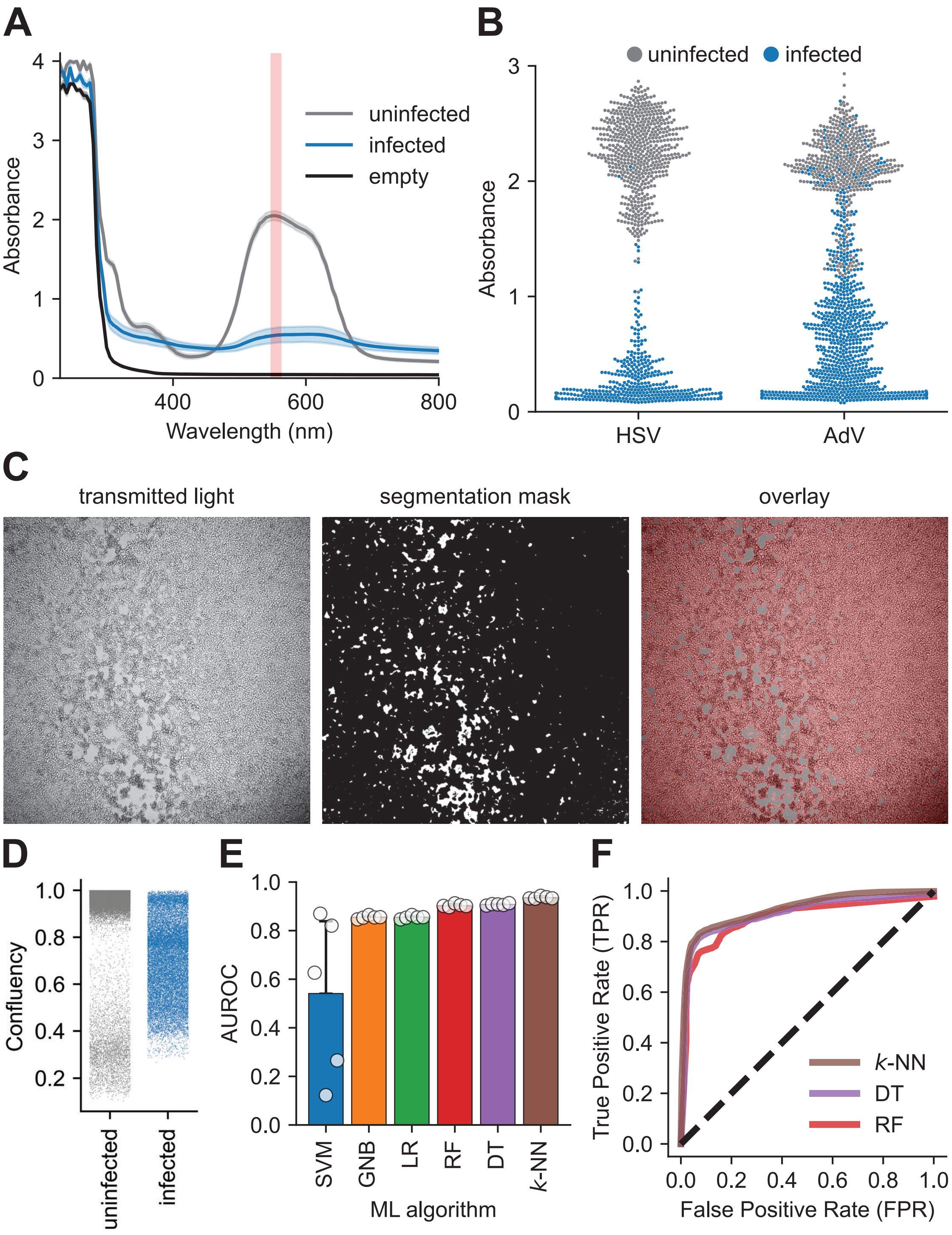

A
4
3
2
1
0
Absorbance
400
600
800
Wavelength (nm)
uninfected
infected
empty
B
uninfected
infected
3
2
1
0
Absorbance
HSV
AdV
C
transmitted light
segmentation mask
overlay
D
1.0
0.8
0.6
0.4
0.2
Confluency
uninfected
infected
E
1.0
0.8
0.6
0.4
0.2
0.0
AUROC
SVM
GNB
LR
RF
DT
k-NN
ML algorithm
F
1.0
0.8
0.6
0.4
0.2
0.0
True Positive Rate (TPR)
0.0
0.2
0.4
0.6
0.8
1.0
False Positive Rate (FPR)
k-NN
DT
RF

Figure S3: Confluency-based image classification

(A) Absorption spectrum of wells stained with crystal violet. The red shaded band around the absorption maximum at 555 ± 4.5 nm was used for subsequent absorbance measurements. Shaded regions around the curves indicate standard deviations. n = 10. **(B)** Absorbance values of A549 cells infected with HSV or AdV. The color indicates evaluation by human experts. HSV: n = 960, AdV: n = 1248. **(C)** Example image for cell segmentation quantification from Figure 1C, showing A549 cells infected with AdV. Confluent regions are shown in black in the segmentation mask, and in red in the overlay. **(D)** Strip plot of all confluency values from the dataset used for subsequent image classification. Uninfected: n = 35,744, infected: n = 22,876. **(E)** Performance characteristics for machine learning models. SVM = Support Vector Machine, GNB = Gaussian Naïve Bayes, LR = Logistic Regression classifier, RF = Random Forest classifier, DT = decision tree classifier, k-NN = k-nearest neighbors. AUROC = area under the receiver operating characteristic curve. Error bars indicate standard deviations, n = 5. **(F)** Receiver operating characteristic (ROC) curves for the three best-performing models. The dotted lines in (E) and (F) indicate a random classifier. Source data are provided as a Source Data file

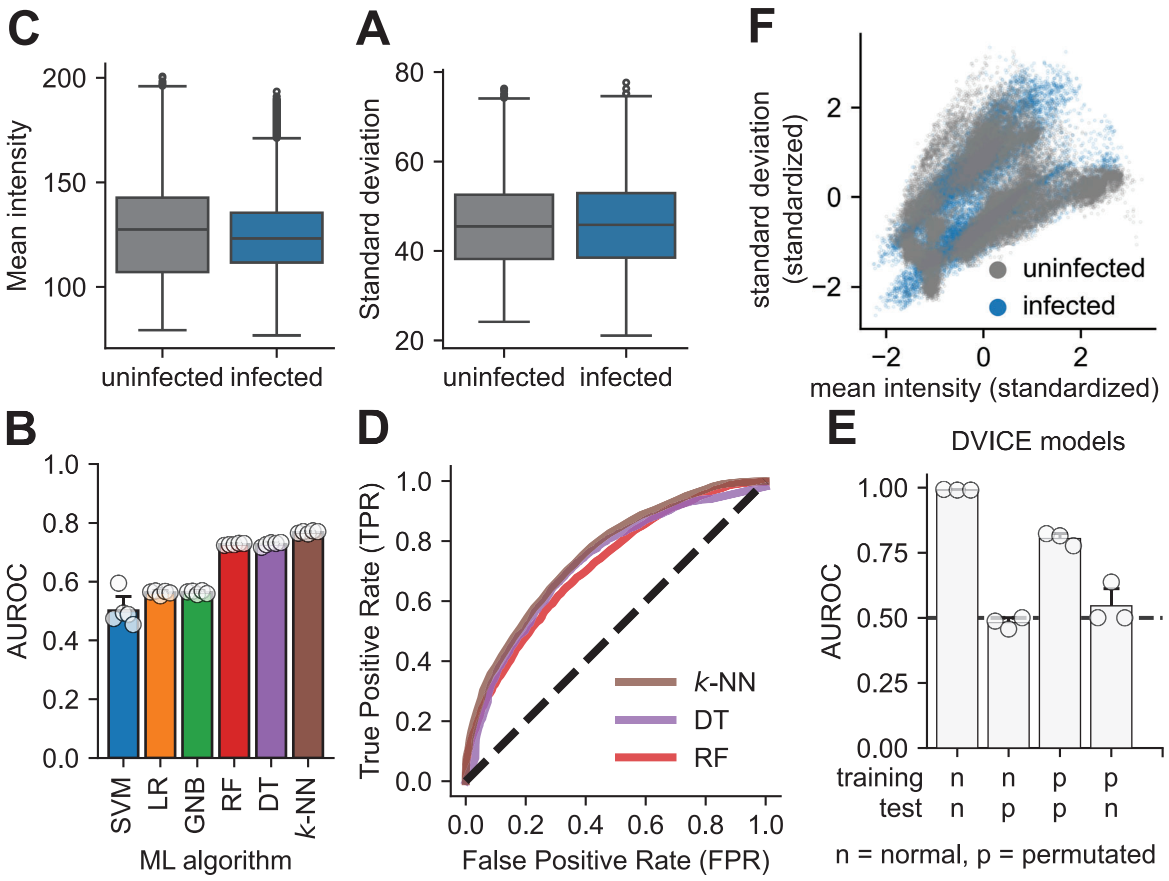
C
Mean intensity
200
150
100
uninfected infected
A
Standard deviation
80
60
40
20
uninfected infected
F
standard deviation (standardized)
mean intensity (standardized)
uninfected
infected
B
AUROC
SVM
LR
GNB
RF
DT
k-NN
ML algorithm
D
True Positive Rate (TPR)
False Positive Rate (FPR)
k-NN
DT
RF
E
DVICE models
AUROC
training
test
n = normal, p = permutated

Figure S4: Intensity-based image classification

(A) Mean intensity of images used in the study. Lines show the medians of the distributions, boxes show the quartiles, whiskers are drawn to the farthest datapoint within 1.5 * inter-quartile range (IQR) from the nearest hinge, and fliers show points extending beyond the whiskers. uninfected: n = 35,746, infected: n = 22,873. **(B)** Standard deviations of image intensities of images used in the study. Lines show the medians of the distributions, boxes show the quartiles, whiskers are drawn to the farthest datapoint within 1.5 * inter-quartile range (IQR) from the nearest hinge, and fliers show points extending beyond the whiskers. n = 35,746, infected: n = 22,873. **(C)** Standardized mean intensity and standard deviation of intensities. Each dot represents one image. uninfected: n = 35,746, infected: n = 22,873. **(D)** Data were split into 90 % training data and 10 % test data in a random, stratified way and machine learning models were trained to classify images into infected or uninfected based on the image intensity and standard deviation provided in C. Data for model training were prepared using a fivefold, stratified shuffle split and optimal model parameters were determined using a grid search. SVM = Support Vector Machine, LR = Logistic Regression classifier, GNB = Gaussian Naïve Bayes, RF = Random Forest classifier, DT = decision tree classifier, *k*-NN = *k*-nearest neighbors. AUROC = area under the receiver operating characteristic curve. n = 5. **(E)** Receiver operating characteristic (ROC) curves for the three best-performing models. The dotted line in (D) and € indicates a random classifier. **(F)** DVICE models were trained on either normal (n) or randomly permutated (p) images. Model evaluation was performed on a withheld test set which also consisted of normal or permutated images. n = 3. Source data are provided as a Source Data file.

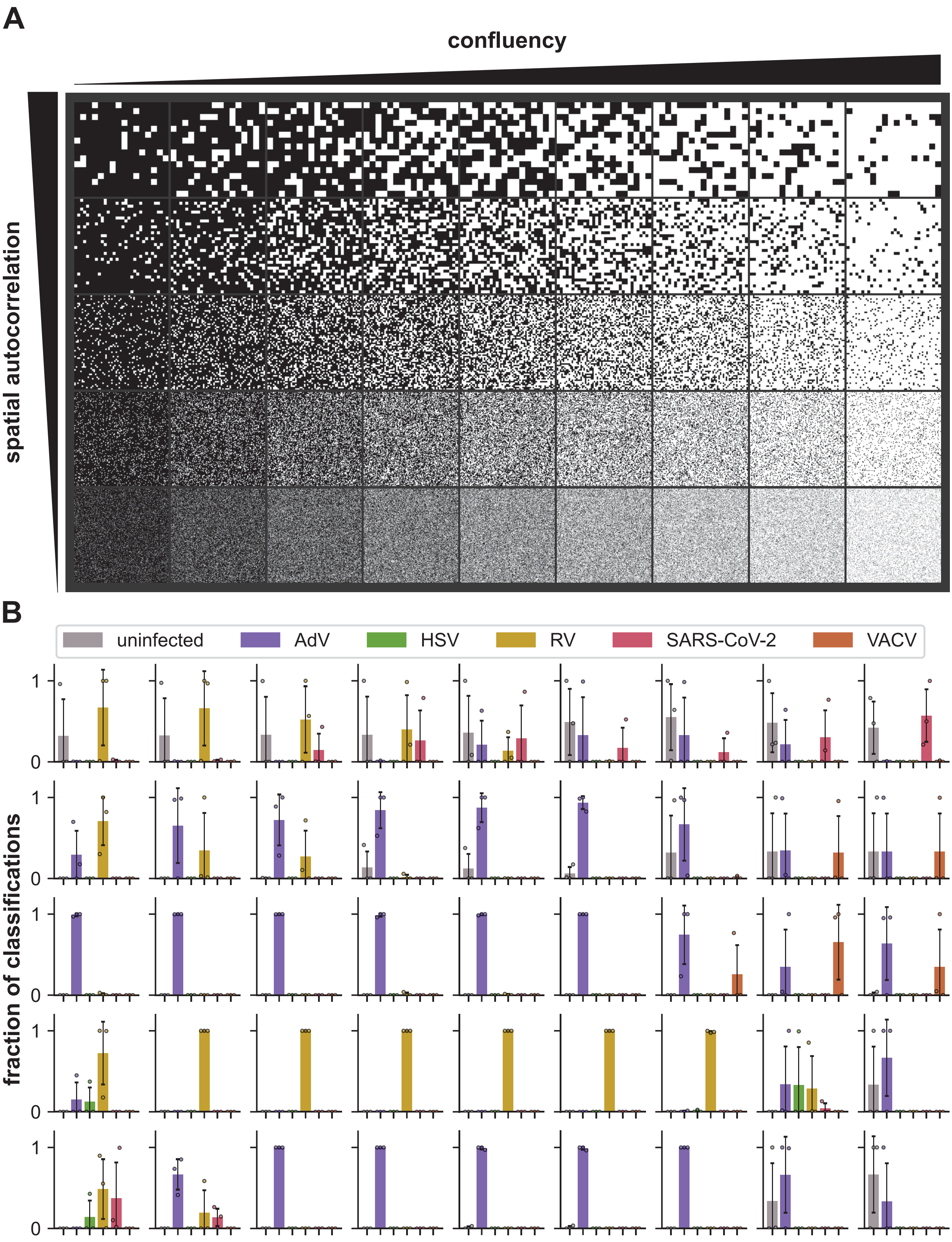

Figure S5: Virus class-specific features

(A) Example images from simulated dataset with varying confluency and spatial autocorrelation. For each category, one thousand images were generated. **(B)** Virus class predictions for simulated dataset. Subpanels refer to the example images shown in panel A. n = 3. Source data are provided as a Source Data file.
