# Supplementary material for: A versatile automated pipeline for quantifying virus infectivity by label-free light microscopy and artificial intelligence
Source: Nat Commun. 2024 Jun 15;15:5112. doi: 10.1038/s41467-024-49444-1 (PMC11180103; doi:10.1038/s41467-024-49444-1)
Supplement: Supplementary file 3 — Reporting Summary [file 41467_2024_49444_MOESM3_ESM.pdf]

nature portfolio

Corresponding author(s): Urs Greber

Last updated by author(s): Apr 25, 2024

Reporting Summary

Nature Portfolio wishes to improve the reproducibility of the work that we publish. This form provides structure for consistency and transparency in reporting. For further information on Nature Portfolio policies, see our Editorial Policies and the Editorial Policy Checklist.

Statistics

For all statistical analyses, confirm that the following items are present in the figure legend, table legend, main text, or Methods section.

n/a	Confirmed	
☐	☒	The exact sample size (*n*) for each experimental group/condition, given as a discrete number and unit of measurement
☐	☒	A statement on whether measurements were taken from distinct samples or whether the same sample was measured repeatedly
☐	☒	The statistical test(s) used AND whether they are one- or two-sided<br>*Only common tests should be described solely by name; describe more complex techniques in the Methods section.*
☒	☐	A description of all covariates tested
☐	☒	A description of any assumptions or corrections, such as tests of normality and adjustment for multiple comparisons
☐	☒	A full description of the statistical parameters including central tendency (e.g. means) or other basic estimates (e.g. regression coefficient) AND variation (e.g. standard deviation) or associated estimates of uncertainty (e.g. confidence intervals)
☐	☒	For null hypothesis testing, the test statistic (e.g. F, t, r) with confidence intervals, effect sizes, degrees of freedom and P value noted<br>*Give P values as exact values whenever suitable.*
☒	☐	For Bayesian analysis, information on the choice of priors and Markov chain Monte Carlo settings
☒	☐	For hierarchical and complex designs, identification of the appropriate level for tests and full reporting of outcomes
☐	☒	Estimates of effect sizes (e.g. Cohen's *d*, Pearson's *r*), indicating how they were calculated

Our web collection on statistics for biologists contains articles on many of the points above.

Software and code

Policy information about availability of computer code

Data collection	Data was acquired at ImageXpress Micro Confocal High-Content Imaging System (Molecular Devices) using MetaXpress version 6.2.3.733
Data analysis	All data was analyzed with python (version 3.9.7). Further information can be found in the manuscript

For manuscripts utilizing custom algorithms or software that are central to the research but not yet described in published literature, software must be made available to editors and reviewers. We strongly encourage code deposition in a community repository (e.g. GitHub). See the Nature Portfolio guidelines for submitting code & software for further information.

Data

Policy information about availability of data

All manuscripts must include a data availability statement. This statement should provide the following information, where applicable:

- Accession codes, unique identifiers, or web links for publicly available datasets
- A description of any restrictions on data availability
- For clinical datasets or third party data, please ensure that the statement adheres to our policy

The imaging data are available under restricted access due to legal considerations involving a patent application by the University of Zurich. Access can be obtained by contacting the lead contacts, Prof. Dr. Urs Greber (urs.greber@mls.uzh.ch) and Dr. Anthony Petkidis (anthony.petkidis@uzh.ch) and will be provided within four weeks for academic use of data and restricted to the particular institution that requested access. Source data are provided with this paper.

Research involving human participants, their data, or biological material

Policy information about studies with human participants or human data. See also policy information about sex, gender (identity/presentation), and sexual orientation and race, ethnicity and racism.

Reporting on sex and gender	For the analysis of infectious particle stability in human saliva, saliva was collected from a healthy, 62-year-old donor of male sex and European origin.
Reporting on race, ethnicity, or other socially relevant groupings	see above
Population characteristics	see above
Recruitment	voluntary donors recruited within the research group who wanted to participate in this study. Participants were not compensated for their participation. Self-selection bias cannot be ruled out but is unlikely to have influenced the results of the study, given the chemical simplicity of the studied material.
Ethics oversight	ethical board of the Canton Zurich, Switzerland (BASEC number Req-2022-01020).

Note that full information on the approval of the study protocol must also be provided in the manuscript.

Field-specific reporting

Please select the one below that is the best fit for your research. If you are not sure, read the appropriate sections before making your selection.

[x] Life sciences   [ ] Behavioural & social sciences   [ ] Ecological, evolutionary & environmental sciences

For a reference copy of the document with all sections, see nature.com/documents/nr-reporting-summary-flat.pdf

Life sciences study design

All studies must disclose on these points even when the disclosure is negative.

Sample size	The sample size was chosen to include at least 5000 images of each infection state in the test data set. No sample size calculation was performed. The total number of images was chosen to correspond to 100 96-well plates (assuming an infection index of 50 %), which corresponds to a large-scale experiment.
Data exclusions	Blurry images were excluded.
Replication	The experimental data set was acquired over an extended period of time. It included 37 distinct time points, which all presented biological replicates of infection assays. Bootstrapping and re-sampling were used to ensure reproducibility. Further validation was performed on an additional imaging device. All attempts of replication were successful.
Randomization	Images were split into training, validation and test data sets randomly, stratified by their infection state. Covariates included the acquisition date and microtiter plate, where it was ensured that all images acquired on a given date would be assigned to the same data set.
Blinding	Group allocation was performed randomly and blinding was not relevant to this study, as class labels were obtained as consensus annotation from three human experts, who performed the annotations independendly.

Reporting for specific materials, systems and methods

We require information from authors about some types of materials, experimental systems and methods used in many studies. Here, indicate whether each material, system or method listed is relevant to your study. If you are not sure if a list item applies to your research, read the appropriate section before selecting a response.

Materials & experimental systems

n/a	Involved in the study	
[x]	[ ]	Antibodies
[ ]	[x]	Eukaryotic cell lines
[x]	[ ]	Palaeontology and archaeology
[x]	[ ]	Animals and other organisms
[x]	[ ]	Clinical data
[x]	[ ]	Dual use research of concern
[x]	[ ]	Plants

Methods

n/a	Involved in the study	
[x]	[ ]	ChIP-seq
[x]	[ ]	Flow cytometry
[x]	[ ]	MRI-based neuroimaging

Eukaryotic cell lines

Policy information about cell lines and Sex and Gender in Research

Cell line source(s)	all indicated in the manuscript, briefly:<br>Monkey: VeroE6, Kindly provided by Dr. Volker Thiel (University of Bern, Switzerland)<br>Monkey: VeroE6-TMPRSS2, Kindly provided by Dr. Volker Thiel (University of Bern, Switzerland) NIBSC 100978<br>Human: Huh7, Kindly provided by Dr. Volker Thiel (University of Bern, Switzerland)<br>Human: Huh7-ACE2, laboratory-made by stable transfection with a lentivector (pLVX-ACE2-IRES-BSD)<br>Human: HeLa, American Type Culture Collection (ATCC)<br>Human: HeLa Ohio, obtained from Laurent Kaiser, Central Laboratory of Virology, University Hospital Geneva, Switzerland ECACC 84121901<br>Human: A549, American Type Culture Collection (ATCC) ATCC CCL-185<br>Human: A549-ACE2, laboratory-made by stable transfection with a lentivector (pLVX-ACE2-IRES-BSD)
Authentication	Cell lines were obtained from trusted sources. No authentication was performed.
Mycoplasma contamination	negative
Commonly misidentified lines<br>(See ICLAC register)	Commonly misidentified cell lines used in this study include HeLa cells, which are an important infection model for RV, VACV, and other viruses.

Plants

Seed stocks	na
Novel plant genotypes	na
Authentication	na
